# A Clinician's Guide to the Treatment of Endometriosis with Elagolix

**DOI:** 10.1089/jwh.2019.8096

**Published:** 2021-04-19

**Authors:** Nicholas Leyland, Stephanie J. Estes, Bruce A. Lessey, Arnold P. Advincula, Hugh S. Taylor

**Affiliations:** ^1^ Department of Obstetrics and Gynecology, McMaster University, Hamilton, Ontario, Canada.; ^2^ Department of Obstetrics and Gynecology, Penn State Health, Hershey, Pennsylvania, USA.; ^3^ Wake Forest Health, Center for Fertility, Endocrine and Menopause, Winston-Salem, North Carolina, USA.; ^4^ Department of Obstetrics and Gynecology, Columbia University Medical Center/New York-Presbyterian Hospital, New York, New York, USA.; ^5^ Department of Obstetrics, Gynecology and Reproductive Sciences, Yale School of Medicine, New Haven, Connecticut, USA.

**Keywords:** endometriosis, elagolix, dysmenorrhea, pelvic pain, GnRH receptor antagonist

## Abstract

Pain associated with endometriosis is a considerable burden for women, permeating all aspects of their lives, from their ability to perform daily activities to their quality of life. Although there are many options for endometriosis-associated pain management, they are often limited by insufficient efficacy, inconvenient routes of administration, and/or intolerable side effects. Elagolix, a nonpeptide, small-molecule gonadotropin-releasing hormone (GnRH) receptor antagonist, is the first new oral therapy to be approved for the treatment of endometriosis-associated pain in the United States in more than a decade. Modulation of estradiol with elagolix is dose dependent and ranges from partial to full suppression. Clinical evidence has shown that elagolix at both approved doses (150 mg once daily and 200 mg twice daily) is effective for reducing symptoms of pelvic pain (dysmenorrhea, nonmenstrual pelvic pain, and dyspareunia), improving quality of life, and decreasing use of rescue analgesics (nonsteroidal anti-inflammatory drugs and/or opioids). The availability of two dosing options allows for individualization of treatment based on baseline clinical factors and response to therapy. Elagolix is well tolerated, with less pronounced hypoestrogenic effects compared with GnRH agonists. This review provides an overview of elagolix, highlighting currently available treatment options and the application of this new treatment for women with endometriosis-associated pain.

## Introduction

Endometriosis is an estrogen-dependent, inflammatory condition marked anatomically by the presence of extrauterine lesions containing endometrial glands and stroma. Affecting an estimated 6%–10% of women, endometriosis is one of the most common gynecologic conditions among reproductive-age women.^1,2^ The symptoms of endometriosis have a tremendous impact on patients' lives, negatively influencing quality of life, emotional well-being, intimate relationships, work life, and daily activities.^3–6^ Endometriosis-associated pelvic pain, which often manifests as dysmenorrhea, nonmenstrual pelvic pain (NMPP), and dyspareunia,^2,7–9^ plays a dominant role in the effect of endometriosis on patients' daily lives and physical functioning.^3,5^

A plethora of pharmaceutical agents are prescribed for management of pain associated with endometriosis, although few of these have been approved for this indication by regulatory bodies.^10^ Among commonly used off-label agents are combined hormonal contraceptives. Findings from a recent meta-analysis revealed that, whereas evidence suggests combined hormonal contraceptives reduce endometriosis-associated pain, supportive data are low quality and there are insufficient data comparing these agents with other options for managing endometriosis-associated pain.^11^ Moreover, combined oral contraceptives are not effective for symptom relief in as many as one-third of symptomatic women with endometriosis, and efficacy may wane over time.^12,13^

Progestin-only formulations have been shown to reduce endometriosis-associated pain,^14^ but are subject to the same progesterone resistance that limits the effectiveness of combined hormonal contraceptives.^12^ Progestin therapy (progestin only oral or depot contraception) is commonly associated with breakthrough bleeding^15^ that could exacerbate symptoms of pain.^16^

In addition, long-term administration of the progestin medroxyprogesterone acetate is linked to decreases in bone mineral density (BMD),^10^ which may not be completely reversible.^17^ In the United States, Food and Drug Administration (FDA)-approved treatment options for endometriosis-associated pain management include three gonadotropin-releasing hormone (GnRH) agonists (leuprolide acetate, goserelin acetate, and nafarelin acetate), two progestins (depot medroxyprogesterone acetate and norethindrone acetate), danazol (which is not commonly prescribed due to its androgenic side effects^1^), and the recently marketed GnRH receptor antagonist, elagolix.^10^

Elagolix is the first orally administered FDA-approved treatment option for endometriosis-associated pain in more than 10 years. Unlike GnRH agonists, which induce a hypoestrogenic state through complete suppression of the hypothalamic-pituitary-ovarian axis, GnRH antagonists such as elagolix partially suppress estradiol, thereby lessening hypoestrogenic side effects (*e.g.*, hot flush, vaginal dryness, reduced BMD, and lipid changes), while maintaining therapeutic efficacy.^18^ Given this mechanistic difference, registration trials of elagolix were conducted without add-back hormonal therapy, which is typically administered in conjunction with GnRH agonists to mitigate hypoestrogenic effects.^1,14^

Another differentiator for GnRH antagonists is that they do not cause the initial GnRH stimulation or “flare” effect observed with GnRH agonists, wherein symptoms may worsen during the first 1–2 weeks of treatment.^10,18^ Elagolix-mediated estradiol suppression is observed within hours of the first dose and is dose dependent, with partial suppression (median estradiol concentration of ∼42 pg/mL) observed with a daily dose of 150 mg and maximum estradiol suppression (estradiol concentration approaching the lower limit of quantification [∼12 pg/mL]) attained at doses of 200 mg twice daily or higher.^19–21^

In clinical trials, elagolix has been shown to reduce pelvic pain (including dysmenorrhea, NMPP, and dyspareunia), improve quality of life, and decrease the need for rescue analgesics in women with endometriosis-associated pain.^22–26^ These improvements were maintained during 12 months of treatment.^27^ As a newer treatment option, clinicians may be unfamiliar with elagolix and its application in clinical practice. Although there are dissenting views,^28^ recent studies support the use of this new, orally active modality.^29–33^ The aim of this review is to provide a practical guide for use of elagolix based on available evidence and clinical experience. It is anticipated that with ongoing experience, expertise will continue to develop regarding the best use of pharmaceutical agents for the management of endometriosis symptomatology.

## Search Methodology

The MEDLINE database and relevant medical/scientific congresses were searched using the term “elagolix” for English language articles or presentations of data from randomized controlled trials or long-term extension studies investigating the efficacy, safety, tolerability, pharmacokinetics, and/or clinical use of elagolix. Reference lists of published articles identified during the literature search were also reviewed for relevant publications or presentations. Guidance on the use of elagolix for the management of endometriosis-associated pain was derived from prescribing information, clinical data, and professional experience, and includes suggestions that are not within the FDA-approved indication and clinical use scenarios where additional data are needed.

## Patient Selection

### Diagnosis

Historically, a definitive diagnosis of endometriosis required laparoscopic lesion visualization and excision with histologic confirmation of endometrial gland, endometrial stroma, and/or hemosiderin-laden macrophage presence. However, even in the absence of a definitive diagnosis, many professional society treatment recommendations permit the use of empiric therapy when there is a strong suspicion or clinical evidence of endometriosis.^1,34–36^ For example, guidelines from the American College of Obstetricians and Gynecologists (ACOG) give a Level B recommendation to empiric therapy with a 3-month course of a GnRH agonist in women with chronic pelvic pain who have undergone an appropriate pretreatment evaluation and in whom oral contraceptives and nonsteroidal anti-inflammatory drugs (NSAIDs) have failed, noting the patient should be informed that treatment response does not confirm an endometriosis diagnosis.^1^

The criteria for suspecting endometriosis differ among guidelines, but generally include the presence of pelvic pain symptoms (progressively worsening dysmenorrhea [particularly when severe and unresponsive to NSAIDs and/or hormonal-based therapies], NMPP, and deep dyspareunia), which may be augmented by physical findings (*e.g.*, pelvic tenderness and nodularity on palpation of the uterosacral ligaments and rectovaginal septum), imaging results, and/or cyclical nongynecologic symptoms (dyschezia, dysuria, hematuria and rectal bleeding, diarrhea/irritable bowel syndrome, and shoulder pain).^34–37^ When symptoms are cyclic in nature and progressive over time, endometriosis is by far the most likely clinical diagnosis.

It is increasingly recognized that identification of endometriosis can be achieved with reasonable certainty using nonsurgical methodologies (*i.e.*, a clinical diagnosis), which include evaluation of pelvic pain symptoms, patient history, physical examination findings, and appropriately performed imaging studies.^38–41^ Although there has yet to be identified a replacement test or tests for diagnostic surgery,^42,43^ clinicians with expertise in endometriosis diagnosis and management have suggested that symptoms, patient history, and clinical assessments taken in totality provide a basis for clinical diagnosis.^39,40^

The benefits of undertaking a clinical diagnosis approach are that it does not require surgery and the attendant risks thereof, utilizes assessment tools that are readily available to most clinicians, and may reduce the delay frequently observed in endometriosis diagnosis. In clinical practice, presumptive diagnosis of endometriosis is often made without laparoscopy or other surgical procedure^2,44^ and can facilitate early and effective management of patients' symptoms.

### Characteristics of patients enrolled in elagolix clinical trials

Pivotal elagolix clinical trials enrolled 1689 premenopausal adult women (ages 18–49 years) who had been diagnosed with endometriosis through laparoscopy or laparotomy within the past 10 years and were currently experiencing moderate-to-severe endometriosis-associated pain.^26^ The extent of endometriosis-associated pain during the screening interval was evaluated by Composite Pelvic Signs and Symptoms Score and daily electronic diary entries, in which patients recorded the frequency and severity of overall endometriosis-associated pain (using the numeric rating scale), dysmenorrhea, NMPP, and dyspareunia, as well as rescue medication use. Study entry criteria required dysmenorrhea to be at least moderate in severity and also be accompanied by at least moderate NMPP.^45^

Given the mechanism of action for elagolix, the study did not include women with a history of nonresponse to GnRH agonists or antagonists, depot medroxyprogesterone acetate, or aromatase inhibitors.^26^ Patients with a history of osteoporosis or other metabolic bone disease were also excluded. Screening dual-energy X-ray absorption scans for BMD of the lumbar spine, femoral neck, or total hip could not be 1.5 or more standard deviations below normal (*i.e.*, *Z* score ≤ −1.5).

Mean time since diagnosis of endometriosis in the study population was ∼3.5–4 years. Mean baseline scores across treatment groups for dysmenorrhea (scale, 0 [no pain] to 3 [severe pain]), NMPP (scale, 0–3), dyspareunia (scale, 0–3), and numeric rating scale (scale, 0 [no pain] to 10 [worst pain ever]) were 2.2, 1.6, 1.5, and 5.6, respectively.^26^ At baseline, the majority of patients (>90%) used analgesic agents (NSAIDs, opioids, or both) for the management of endometriosis-associated pain. Notably, reductions in dysmenorrhea and NMPP during study treatment with elagolix were not influenced by patient age, body mass index (BMI), time since diagnosis, or baseline dysmenorrhea score.^46^ The pivotal elagolix clinical trials were predominantly white (88%), which limits the ability to draw conclusions for other racial groups due to the small sample size.^26^

### Recommendations for patient selection

Whereas in clinical trials elagolix was studied in women with moderate-to-severe pain associated with surgically diagnosed endometriosis, our clinical experience as experts who manage endometriosis coupled with guidance regarding empiric therapy suggests that elagolix may be appropriate for use in a wide range of women with endometriosis. For patients who have failed first-line therapy, either as empiric treatment or after a surgical diagnosis of endometriosis, elagolix offers an additional oral option. Similarly, women with progestin-resistant disease, who would otherwise transition to a GnRH agonist or other second-line therapy, may now benefit from elagolix.

As an initial therapy, elagolix may be considered for women who present with very severe endometriosis-associated pain and/or severe endometriosis-related dyspareunia, and in whom other causes of pelvic pain have been ruled out. It may also be an appropriate first-line therapy for those with a history of side effects (mood changes, bloating, and breast tenderness) from contraceptive agents.

Elagolix is not recommended for patients with a history of nonresponse to GnRH agonists or antagonists, and is contraindicated in women who are pregnant, have known osteoporosis, or have severe hepatic impairment. Elagolix should not be used concomitantly with strong organic anion-transporting polypeptide 1B1 inhibitors (*e.g.*, cyclosporine and gemfibrozil). A summary of recommendations for patient selection, including additional treatment considerations, is presented in Table 1.

**Table 1.** Identifying Patients Who May Benefit from Elagolix

Candidates for Elagolix^a^
Premenopausal women with surgically or clinically diagnosed endometriosis, who:
Have endometriosis-associated pain,
Have not responded to or have intolerable side effects with first-line treatments (*e.g.*, NSAIDs, combined hormonal contraceptives, and progestins),
Have progestin-resistant disease, and/or
Have had side effects from oral contraceptive in the past

Considerations/cautions
Not recommended for patients with a history of or nonresponse to GnRH agonists or antagonists
Limit concomitant use with strong CYP3A inhibitors (≤1 month with elagolix 200 mg twice daily and ≤6 months with elagolix 150 mg once daily)
Clinical monitoring is recommended when coadministered with digoxin
Concomitant use with rifampin is not recommended with elagolix 200 mg twice daily and should be limited to 6 months with elagolix 150 mg once daily
Dose increases of midazolam and rosuvastatin may be considered when used concomitantly with elagolix

Contraindications
Pregnancy
Known osteoporosis
Severe hepatic impairment (Child-Pugh C)
Concomitant use of strong OATP1B1 inhibitors (*e.g.*, cyclosporine and gemfibrozil)

^a^ These criteria reflect expert opinion. Pivotal elagolix clinical trials enrolled patients with a surgical diagnosis of endometriosis and who were experiencing moderate-to-severe endometriosis-associated pain; treatment history was not a factor for study eligibility.

CYP, cytochrome P450; GnRH, gonadotropin-releasing hormone; NSAIDs, nonsteroidal anti-inflammatory drugs; OATP, organic anion-transporting polypeptide.

## Elagolix Dosing

**FIG. 1.**
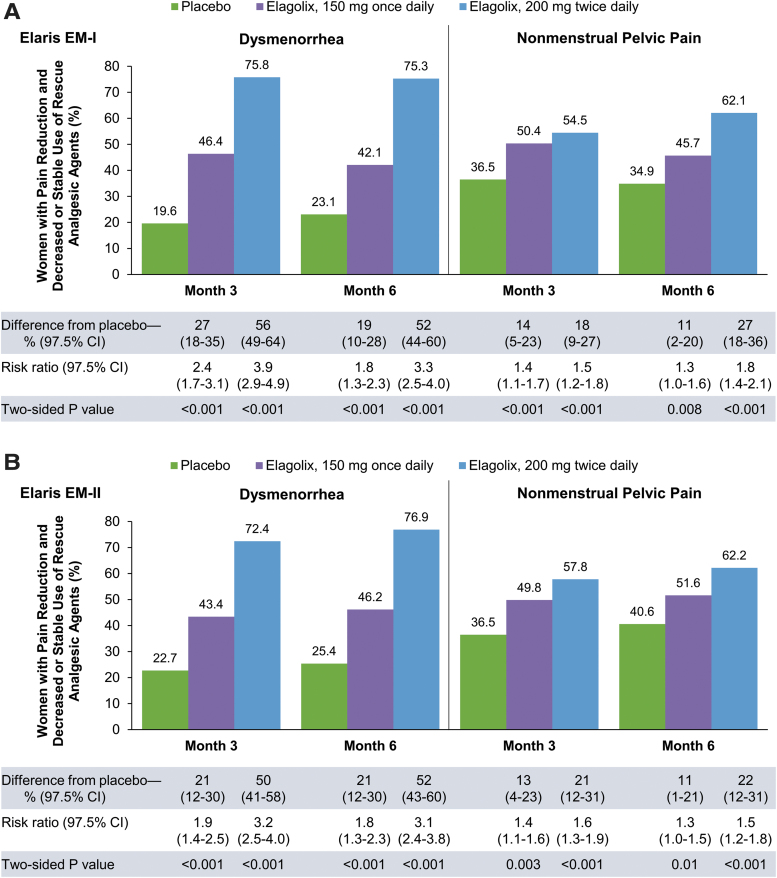
Response rates among women with moderate-to-severe endometriosis-associated pain in two phase 3, placebo-controlled clinical trials of elagolix. Primary endpoint results from the Elaris EM-I **(A)** and Elaris EM-II **(B)** clinical trials. Clinical response was defined as a clinically meaningful reduction in pain score and decreased or stable use of rescue analgesic agents. Thresholds for a clinically meaningful change from baseline were −0.81 for dysmenorrhea and −0.36 for NMPP in Elaris EM-I and −0.85 for dysmenorrhea and −0.43 for NMPP in Elaris EM-II. *p*-Values are for the comparison of each elagolix treatment group versus placebo. Reproduced with permission from Taylor et al.^26^ CI, confidence interval.

Dosing regimens for elagolix are consistent with the pivotal clinical trials: 150 mg once daily and 200 mg twice daily. Both doses were effective for improving dysmenorrhea and NMPP, with significantly greater proportions of women demonstrating a clinically meaningful reduction in each pain symptom in association with decreased or stable use of rescue analgesics compared with placebo (*p* < 0.001; Fig. 1) at month 3, the coprimary efficacy endpoints in the pivotal phase 3 clinical trials.^26^ Decreases in endometriosis-associated pain were dose dependent and maintained throughout the treatment period.

**FIG. 2.**
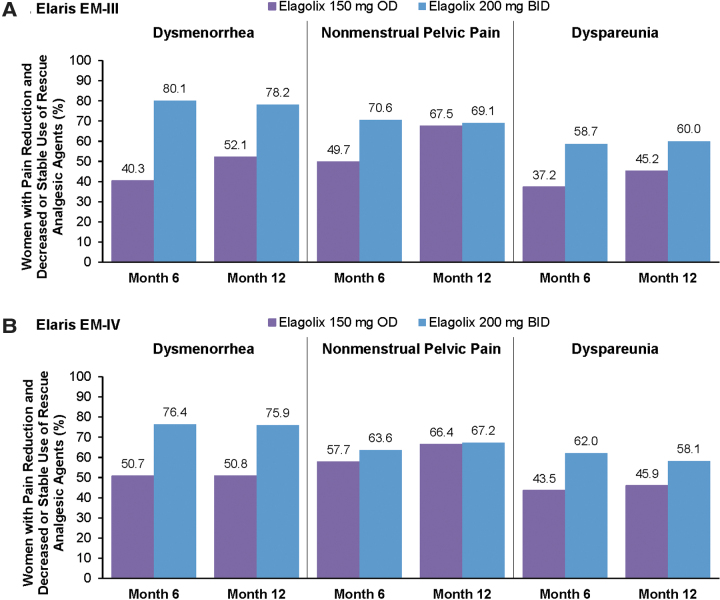
Response rates in two phase 3, long-term extension studies of elagolix for moderate-to-severe endometriosis-associated pain. Proportion of dysmenorrhea, NMPP, and dyspareunia responders in the Elaris EM-III **(A)** and Elaris EM-IV **(B)** clinical trials. Clinical response was defined as patients who experienced a clinically meaningful reduction in the respective type of pain (using the same thresholds as determined in the placebo-controlled trials)^26^ and decreased or stable rescue analgesic use. Data from Surrey et al.^27^ BID, twice daily; OD, once daily.

Patients who completed study treatment in the elagolix treatment arms of the pivotal trials were eligible for continued treatment at the same dose in companion extension studies. Rates of study completion in the pivotal trials were comparable at both elagolix doses and slightly higher compared with placebo (79% for elagolix 150 mg once daily, 77% for elagolix 200 mg twice daily, and 74% for placebo). During long-term extension studies, which increased total treatment duration to 12 months, sustained or improved reductions in dysmenorrhea, NMPP, and dyspareunia were observed (Fig. 2).^27^

Treatment with elagolix should be initiated at the lowest effective dose. Although for most patients, the lowest effective starting dose will be 150 mg once daily, there are scenarios where beginning treatment with the higher dosing regimen is appropriate. For example, initiating therapy with elagolix 200 mg twice daily is recommended for patients in whom dyspareunia is the main symptom and may be considered for patients with severe NMPP or a history of endometriosis pain severe enough to require opioid analgesics. These recommendations are based, in part, on a greater reduction in rescue opioid use and improvement in dyspareunia at month 3, key secondary efficacy endpoints in pivotal elagolix clinical trials, with the higher-dose regimen (Table 2).^26^ For the elagolix 150 mg once-daily regimen, a numeric reduction in dyspareunia score was observed, but the comparison versus placebo did not achieve statistical significance for dyspareunia score reduction or decrease in use of rescue analgesics. At their discretion, physicians may also choose to initiate therapy at the 200 mg twice-daily dose due to symptom severity or clinical history, and subsequently transition to the 150 mg once-daily dose.

**Table 2.** Reduction in Rescue Medication Use and Improvement in Dyspareunia by Elagolix Dose in Two Placebo-controlled Phase 3 Clinical Trials

Parameter	Elaris EM-I			Elaris EM-II		
	Placebo	Elagolix, 150 mg once daily	Elagolix, 200 mg twice daily	Placebo	Elagolix, 150 mg once daily	Elagolix, 200 mg twice daily
Score for dyspareunia^a^						
At 3 months						
No. of women	246	171	153	226	145	150
Change in score	–0.29 ± 0.04	–0.39 ± 0.05	–0.49 ± 0.05	–0.30 ± 0.04	–0.39 ± 0.05	–0.60 ± 0.05
Difference from placebo	—	–0.09 ± 0.07	–0.20 ± 0.07^b^	—	–0.09 ± 0.07	–0.30 ± 0.07^c^
Use of rescue analgesic agent^d^						
At 3 months						
No. of women	329	226	213	312	204	209
Change in score	–0.29 ± 0.03	–0.29 ± 0.04	–0.55 ± 0.04	–0.31 ± 0.03	–0.36 ± 0.04	–0.49 ± 0.03
Difference from placebo	—	–0.01 ± 0.05	–0.26 ± 0.05^c^	—	–0.05 ± 0.04	–0.18 ± 0.04^c^
At 6 months						
No. of women	288	198	182	273	185	187
Change in score	–0.27 ± 0.04	–0.35 ± 0.04	–0.56 ± 0.05	–0.32 ± 0.03	–0.40 ± 0.04	–0.52 ± 0.04
Difference from placebo	—	–0.07 ± 0.06	–0.28 ± 0.06^c^	—	–0.08 ± 0.05	–0.21 ± 0.05^c^
Use of rescue opioid^d^						
At 3 months						
No. of women	329	226	213	312	204	209
Change in score	–0.10 ± 0.02	–0.07 ± 0.03	–0.22 ± 0.03	–0.12 ± 0.02	–0.12 ± 0.02	–0.21 ± 0.02
Difference from placebo	—	0.03 ± 0.04	–0.12 ± 0.04^b^	—	0.00 ± 0.03	–0.08 ± 0.03^b^

Reproduced with permission from Taylor et al.^26^ Data are least-squares means ± SE.

^a^ Pain scores range from 0 (none) to 3 (severe) and were recorded in a daily electronic diary. Scores on the scale for dyspareunia were analyzed for women who recorded data other than “not applicable” at baseline and at one or more measurements after baseline.

^b^ *p* < 0.01.

^c^ *p* < 0.001.

^d^ The use of rescue NSAIDs or opioids was based on average pill counts.

SE, standard error.

Elagolix dose determinations are not influenced by body weight/BMI or by presence of renal impairment, end-stage renal disease, or mild hepatic impairment (as indicated by a prognostic categorization for chronic liver disease/cirrhosis of Child-Pugh A).^21^ Due to increased drug exposures in women with moderate or severe hepatic impairment compared with those who have normal hepatic function, treatment should be limited to 6 months of elagolix 150 mg once daily for women with moderate hepatic impairment (Child-Pugh B), and is contraindicated in patients with severe hepatic impairment (Child-Pugh C).

Patients starting elagolix should expect a decrease in endometriosis-associated pain within 4 weeks of initiating treatment. In both pivotal clinical trials, significant reductions in dysmenorrhea (∼40%–50% reduction with elagolix vs. <20% reduction with placebo; *p* ≤ 0.001) and NMPP (∼20% reduction with elagolix vs. ∼10% reduction with placebo; *p* < 0.05 for elagolix 200 mg twice daily) versus placebo were observed at 1 month after beginning elagolix.^26^ A follow-up assessment of response to therapy and tolerability should be conducted within 3 months or less of starting elagolix. Patients should be queried about the severity and frequency of individual symptoms, ability to perform daily activities at work and in the home, and improvement in their quality of life, as well as compliance with treatment.

For individuals who report improvement with elagolix 150 mg once daily but continue to experience endometriosis-associated pain that interferes with daily life or activities, switching to elagolix 200 mg twice daily may be appropriate. Conversely, if pain relief is sufficient with elagolix 200 mg twice daily, but is accompanied by hypoestrogenic side effects, the 150 mg once-daily dose may be preferred. The use of elagolix 150 mg twice daily has not been studied; however, in patients receiving the 150-mg once-daily dose who require a dose increase, this intermediate dose may offer a practical transition step to elagolix 200 mg twice daily.

The use of add-back therapy to alleviate hypoestrogenic side effects has not been established, but is currently under investigation (see Managing Endometriosis as a Chronic Disease: Long-Term Treatment Considerations). Until these results are available, clinicians should consider use of add-back therapy as they would for other conditions that lead to vasomotor symptoms.

## Safety Considerations

Consistent with the mechanism of action, hypoestrogenic effects are among the most common adverse events reported during elagolix clinical trials. As this was an anticipated effect, special attention was given to the occurrence of vasomotor symptoms and to changes in BMD, lipids, and endometrial thickness. In the pivotal studies, hot flushes were reported by 24% of women who received elagolix 150 mg once daily and by up to 48% of women who received elagolix 200 mg twice daily.^26^ The majority of hot flushes were mild or moderate in severity, and infrequently resulted in study drug discontinuation (<1% and <3% in the lower- and higher-dose groups, respectively). The frequency of hot flushes with elagolix was lower than that associated with leuprolide acetate, for which rates as high as 84% have been reported.^47^ In a 24-week, phase 2 study, hot flushes occurred at a similar frequency among patients who received elagolix 150 mg once daily or depot medroxyprogesterone acetate.^23^ Patients who are starting an elagolix regimen should be apprised of the potential for experiencing hot flushes.

Dose- and duration-dependent decreases in BMD have been observed in elagolix clinical trials. The magnitude of decrease was generally modest.^23–27^ After 6 months of treatment in phase 3 studies, mean percentage changes in lumbar spine BMD were −0.3% to −0.7% with elagolix 150 mg once daily and −2.5% to −2.6% with elagolix 200 mg twice daily.^27^ Follow-up assessments in a long-term extension study revealed partial recovery of BMD at 6 and 12 months post-treatment,^27^ although the influence of these BMD changes on bone health and fracture risk over time is currently not known.

Decreases in BMD over a 24-week treatment period with elagolix 150 mg once daily were similar to those observed with subcutaneous depot medroxyprogesterone acetate (104 mg/0.65 mL administered on weeks 1 and 12) in a phase 2, randomized, double-blind, head-to-head comparison.^23^ In this study, elagolix was noninferior to depot medroxyprogesterone acetate for reduction in dysmenorrhea and NMPP. Changes in BMD over 6 months of elagolix treatment at either dose were less than has been observed with GnRH agonists (−3.2% to −4.3%).^47–49^ Over a short-term assessment period (12 weeks) in a phase 2 study, decreases in BMD were lower with elagolix 150 or 250 mg once daily compared with leuprolide acetate 3.75 mg.^25^ Patients with risk factors for bone loss or osteoporosis (*e.g.*, history of low-trauma fracture, family history of osteoporosis, and lifestyle risk factors) should undergo BMD assessment before starting elagolix. Elagolix is contraindicated in patients with known osteoporosis (*e.g.*, a BMD T-score of ≤ −2.5). In women with risk factors for bone loss, BMD measurement should be performed after 1 year of treatment if the patient will be continuing with the drug. To promote bone health during treatment, calcium and vitamin D supplementation as well as positive bone health choices are recommended. The use of add-back therapy to preserve BMD when using elagolix for the treatment of endometriosis has not been studied, but is currently under evaluation and may allow continued use of the higher dose.

The hypoestrogenic effects of GnRH modulators include changes in the lipid profile.^10^ With elagolix, dose-dependent increases were observed in total cholesterol, low-density lipoprotein cholesterol, high-density lipoprotein cholesterol, and triglycerides.^26^ Lipid increases occurred primarily during the first 1–2 months of treatment and remained stable thereafter.^21^ A return to the baseline lipid profile occurred within 1 month of discontinuing treatment.^27^ It is unlikely that these small changes are clinically relevant in reproductive-age women.

Other common mild adverse events that occurred during elagolix clinical trials included headache, insomnia, mood swings, night sweats, and arthralgia.^26^ Patients should be made aware that elagolix may decrease menstrual bleeding or cause amenorrhea, thereby obscuring early recognition of pregnancy. Depression and mood changes, particularly if these include suicidal ideation, warrant further investigation, with assessment of the benefits and risks of continuing treatment and referral to a mental health professional, as appropriate. A low level of asymptomatic increases in hepatic aminotransferases has been reported; during the pivotal trials, increases in alanine aminotransferase greater than three times the upper limit of normal occurred in 0.2%, 1.1%, and 0.1% of patients in the elagolix 150 mg once-daily, elagolix 200 mg twice-daily, and placebo groups, respectively.^21^

No adverse effects on the endometrium have been observed with elagolix.^26,27^ Endometrial thickness decreased during 6-month treatment with elagolix, and endometrial biopsy samples showed an increase in the proportion of women with quiescent or minimally stimulated endometrial tissue.^26^

## Addressing the Need for Contraception During Elagolix Use

Pregnancy should be discouraged in women taking elagolix. Ovulation is still possible for some women during elagolix treatment; therefore, patients on elagolix should be advised to use contraception during treatment and for 1 week after completing treatment.^21^ During the clinical development program, 49 pregnancies were reported among women who received elagolix.^21^ Although preclinical data do not suggest that elagolix has teratogenic effects,^23^ 2 congenital malformations occurred among the 49 reported pregnancies.^21^ Overall, the number of pregnancies and duration of exposure are too limited to determine the effect of elagolix on pregnancy.

Prescribing information for elagolix recommends the use of nonhormonal contraceptives during elagolix treatment and for 1 week after discontinuation of therapy. Although drug–drug interactions between elagolix and norethindrone or ethinyl estradiol-containing oral contraceptives are minimal,^50^ data are lacking regarding the effects of concomitant use on efficacy. A phase 3 clinical trial assessing the efficacy and safety of elagolix in combination with combined oral contraceptives is currently ongoing (ClinicalTrials.gov identifier, NCT03213457). Estrogen-containing contraceptives are not recommended in women treated with elagolix, as the introduction of exogenous estrogen may lower treatment efficacy. The potential influence of progestin-only contraceptives on elagolix efficacy is not known; however, progestins may add benefit in controlling endometriosis and may be considered. Similarly, a progestin-releasing intrauterine system may provide contraception and augment treatment of endometriosis. Patients who become pregnant while using elagolix should immediately discontinue therapy.

## Managing Endometriosis as a Chronic Disease: Long-Term Treatment Considerations

Management of endometriosis is an ongoing concern that does not resolve with completion of a single course of therapy; even patients who undergo surgical endometriosis lesion removal have a high likelihood for recurrence of symptoms.^51^ A course of treatment with elagolix in patients with normal liver function is recommended to continue for up to 24 months for the 150 mg once-daily dose and up to 6 months for the 200 mg twice-daily dose.^21^ Although there are no data to support a step-down strategy, it may be feasible to transition from elagolix 200 mg twice daily to elagolix 150 mg once daily for longer-term treatment.

There is currently no clinical evidence to indicate how long after treatment cessation the positive effects of therapy will be maintained. Endometriosis-associated pain often recurs after completion of any therapy regimen.^52^ As with surgery or medical therapy modalities such as leuprolide acetate, repeat dosing with elagolix may be of benefit, especially if well tolerated and efficacious the first time. Clinical studies and experience are needed to assess the benefit to the patient of this approach.

With the availability of generic and low-cost options for managing endometriosis-associated pain, the cost-to-benefit ratio of any emerging treatment is an important consideration. In their 2018 assessment of elagolix, the Institute for Clinical and Economic Review (ICER) reported that “use of elagolix to treat moderate-to-severe endometriosis-related pain provides clinical benefit in terms of gains in health-related quality of life relative to no active treatment.”^53^ In 2019, Wang et al.^54^ published a cost-effectiveness evaluation that indirectly compared elagolix with leuprolide acetate, described as one of the most commonly used second-line therapies for endometriosis. Both elagolix treatment regimens analyzed (150 mg once daily for 24 months and 200 mg twice daily for 6 months) were found to be more cost-effective than leuprolide acetate (11.25 mg every 3 months for 12 months, with add-back norethindrone acetate during the latter 6 months). These data suggest that the cost-to-benefit ratio is generally favorable for elagolix, although additional data in comparison with other treatment options, for treatment durations of greater than 12 months, and in real-world patient populations are needed to fully assess the value of treatment.

It is important to note that all of the clinical data described herein reflect elagolix treatment without add-back therapy, as there are no completed studies to date in which add-back therapy was used in conjunction with elagolix for the management of endometriosis-associated pain. Add-back therapy with progestins alone, progestins and bisphosphonates, and low-dose progestins with estrogens has been shown to reduce the hypoestrogenic effects of GnRH agonists (*e.g.*, hot flushes, bone loss), while maintaining endometriosis-associated pain reduction.^1,14,55^ Add-back therapy also makes it possible to extend the duration of GnRH agonist therapy.^1^

Although add-back therapy data are not yet available in the context of endometriosis, results from a proof-of-concept study in women with uterine fibroids and heavy menstrual bleeding demonstrated that add-back therapy with continuous estradiol 0.5 mg/norethindrone acetate 0.1 mg or continuous estradiol 1 mg and cyclical progestogen 200 mg decreased the occurrence of hot flushes and lessened the effect of elagolix on the lipid profile.^56^ Whether these same reductions in hypoestrogenic effects with add-back therapy will occur in women with endometriosis is being assessed in an ongoing study of elagolix combined with low-dose estradiol/norethindrone acetate (Table 3).

**Table 3.** Clinical Trial of Elagolix with Add-Back Therapy

Phase 3, randomized, double-blind placebo-controlled study
Enrollment: 680 adult women with moderate-to-severe endometriosis-associated pain
Treatment arms:
Elagolix 200 mg BID + low-dose estradiol/norethindrone acetate
Elagolix 200 mg BID
Placebo
Duration: 12 months
Primary outcome measures:
Proportion of responders based on dysmenorrhea at month 6
Proportion of responders based on NMPP at month 6
Secondary outcome measures:
Change from baseline in dysmenorrhea (months 3, 6, and 12)
Change from baseline in dyspareunia (months 3, 6, and 12)
Change from baseline in numeric rating scale (months 3, 6, and 12)
Change from baseline in NMPP (months 3, 6, and 12)

ClinicalTrials.gov identifier: NCT03213457.

BID, twice daily; NMPP, nonmenstrual pelvic pain.

## Summary

Elagolix offers a new option for the management of endometriosis-associated pain. As an oral agent with titratable estradiol suppression, elagolix provides an alternative to injectable agents and those that require add-back therapy to mitigate complete hypothalamic-pituitary-ovarian axis suppression. In addition, the availability of two dosage regimens allows for individualization of treatment based on clinical presentation, clinical response, and tolerability.

Elagolix was approved by the FDA in 2018 for the management of moderate-to-severe pain associated with endometriosis. Currently available data suggest that elagolix may be appropriate for many women, which, in our expert opinion, includes empiric therapy for select patients in accordance with the limitations described by endometriosis management guidelines. The decision regarding whether this is a suitable option for an individual patient should include an assessment of contraindications such as known osteoporosis or severe hepatic impairment and consideration for conditions that might influence treatment efficacy such as a history of nonresponse to GnRH agonists or antagonists. The physician-patient discussion should also include the potential for hypoestrogenic side effects, including vasomotor symptoms and decreases in BMD, as well as use of contraception during elagolix treatment.

Although there are areas for further investigation (*e.g.*, assessment of efficacy and safety in real-world populations, potential for use of add-back therapy to reduce hypoestrogenic side effects, and comparisons with low-dose hormonal contraceptives and progestins), clinical evidence suggests that elagolix is effective and well tolerated in patients with moderate-to-severe endometriosis-associated pain.
